# Kinase Activity of Fission Yeast Mph1 Is Required for Mad2 and Mad3 to Stably Bind the Anaphase Promoting Complex

**DOI:** 10.1016/j.cub.2011.12.049

**Published:** 2012-02-21

**Authors:** Judith Zich, Alicja M. Sochaj, Heather M. Syred, Laura Milne, Atlanta G. Cook, Hiro Ohkura, Juri Rappsilber, Kevin G. Hardwick

**Affiliations:** 1 Wellcome Trust Centre for Cell Biology, University of Edinburgh, King's Buildings, Mayfield Road, Edinburgh, EH9 3JR, UK

## Abstract

Defects in chromosome segregation result in aneuploidy, which can lead to disease or cell death [1, 2]. The spindle checkpoint delays anaphase onset until all chromosomes are attached to spindle microtubules in a bipolar fashion [3, 4]. Mad2 is a key checkpoint component that undergoes conformational activation, catalyzed by a Mad1-Mad2 template enriched at unattached kinetochores [5]. Mad2 and Mad3 (BubR1) then bind and inhibit Cdc20 to form the mitotic checkpoint complex (MCC), which binds and inhibits the anaphase promoting complex (APC/C). Checkpoint kinases (Aurora, Bub1, and Mps1) are critical for checkpoint signaling, yet they have poorly defined roles and few substrates have been identified [6–8]. Here we demonstrate that a kinase-dead allele of the fission yeast *MPS1* homolog (Mph1) is checkpoint defective and that levels of APC/C-associated Mad2 and Mad3 are dramatically reduced in this mutant. Thus, MCC binding to fission yeast APC/C is dependent on Mph1 kinase activity. We map and mutate several phosphorylation sites in Mad2, producing mutants that display reduced Cdc20-APC/C binding and an inability to maintain checkpoint arrest. We conclude that Mph1 kinase regulates the association of Mad2 with its binding partners and thereby mitotic arrest.

## Graphical abstract

**Graphical Abstract:**

**Figure:**
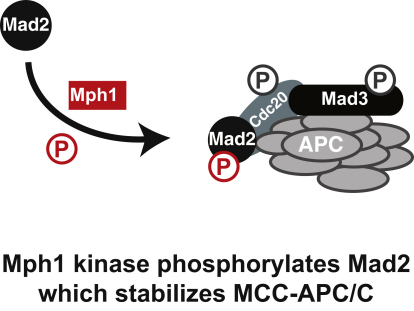

**Highlights:**

► Mph1 kinase activity is required for stable binding of Mad2/3 to Cdc20^Slp1^-APC/C ► Mph1 kinase phosphorylates Mad2 ► *mad2-S92A* has reduced APC/C binding and is unable to maintain checkpoint arrest ► *mad2-S187D* suggests a possible role for Mph1 kinase in checkpoint inhibition

## Results and Discussion

### *mph1-D459A* Is a Kinase-Dead Allele

**Figure 1:**
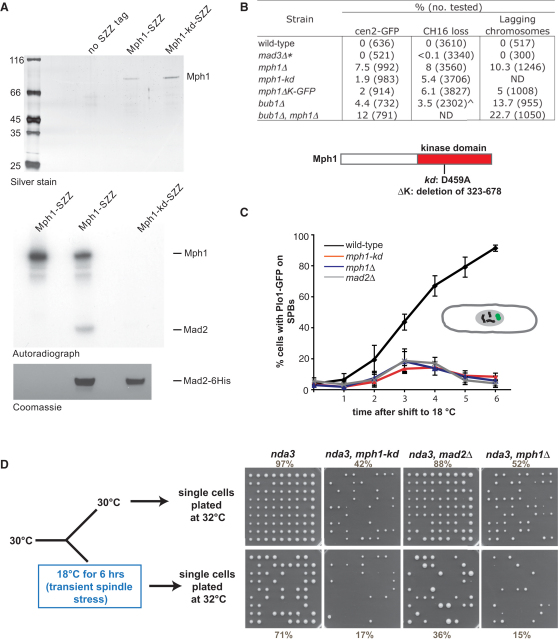
*mph1* Kinase-Dead Mutants Have Both Chromosome Segregation and Spindle Checkpoint Defects (A) In vitro kinase assays demonstrate that *mph1-kd* is kinase dead and that Mph1 kinase autophosphorylates and can phosphorylate Mad2 in vitro. Kinase assays were carried out with Mph1- and Mph1-kd-SZZ proteins purified from fission yeast, with recombinant Mad2 as substrate. (B) Table summarizing chromosome segregation defects of *mph1* alleles. Certain data are taken from published work, ^∗^ on Mad3 [11] and ^∧^ on Bub1 [13]. The schematic indicates the kinase domain and the D459 residue that was mutated to alanine. (C) *mph1-kd* strains fail to arrest in the absence of microtubules. The indicated strains, each containing the cold-sensitive *nda3-KM311* mutation and GFP-tagged Plo1, were shifted to the cold (18°C) for 6 hr. Their mitotic index was scored with Plo1-GFP on spindle poles as a marker. (D) *mph1-kd* cell viability is lost in the absence of microtubules. Strains were grown for 6 hr at 18°C, and then single cells were plated and their ability to form colonies assayed. Numbers indicate percent viability, where n was >240 for each strain. See also Figure S1.

*S. pombe* Mph1 is the homolog of *S. cerevisiae* Mps1, but it is neither required for spindle pole duplication nor essential for cell viability [9]. In other organisms Mps1 kinase is multifunctional, with roles in spindle pole duplication, kinetochore biorientation, mitotic timing, spindle checkpoint signaling, and meiosis [7, 8]. To test whether Mph1 kinase activity was required for distinct functions in fission yeast, we made two “kinase-dead” alleles. In one the whole kinase domain, residues 323–678, was deleted (*mph1ΔK*). Kinase-dead mutants were made in budding yeast [10], and we introduced a similar substitution (D459A) in Mph1 (*mph1-kd*). For some experiments, we added the SZZ tandem affinity purification (TAP) tag to enable rapid and efficient purification from extracts. Affinity-purified Mph1-SZZ kinase was incubated with γ-[^32^P]ATP and recombinant Mad2 substrate (Figure 1A). Several conclusions can be drawn: first, Mph1 is an active kinase that can phosphorylate itself and Mad2 in vitro; second, the D459A substitution has “killed” Mph1 kinase activity; and third, the Mph1-SZZ purification was specific.

### Mph1 Kinase Has Chromosome Segregation Functions

Deletion of many checkpoint (*mad* and *bub*) genes in fission yeast has a modest effect on chromosome loss rate [11, 12], yet abrogation of checkpoint kinases (Bub1 and Ark1) has a profound effect [13, 14]. *mph1Δ* and kinase-dead alleles are very sensitive to antimicrotubule drugs, displaying a similar phenotype to *bub1Δ* (Figure S1A available online). We quantitated their chromosome loss rates with three distinct assays: analysis of the mitotic segregation of GFP-marked chromosome 2; colony-sectoring analysis of the rate of loss of the mini-chromosome Ch16; and analysis of lagging chromosomes in anaphase cells (Figures 1B and S1B–S1D). *mph1-kd* alleles have significant chromosome loss rates, although lower than *mph1Δ.* These experiments demonstrate that Mph1 kinase has important chromosome segregation function(s), even when there are no extrinsic perturbations of spindle microtubules.

### *mph1* Kinase-Dead Mutants Are Checkpoint Defective

The benomyl sensitivity of *mph1* alleles could in part be due to a defective spindle checkpoint, and to test this we analyzed their ability to arrest in mitosis in the absence of microtubules. We employed the *nda3* (β-tubulin) mutant, which arrests at 18°C with hypercondensed chromosomes, no mitotic spindle, and unattached kinetochores. To quantitate mitotic index, we analyzed cells containing Plo1-GFP, which localizes to spindle pole bodies (SPBs) in mitosis. The *nda3* cells were grown to early log phase at 30°C and shifted to 18°C, and their mitotic index was quantitated throughout a 6 hr time course. The *mph1-kd* mutant was unable to arrest in mitosis, like *mad2Δ* and *mph1Δ* strains (Figure 1C). We conclude that Mph1 kinase activity is required for checkpoint arrest. The consequence of this inability to arrest is a rapid loss in viability. To quantitate this, we took *nda3* cultures, shifted them to 18°C for 6 hr, then isolated single cells and plated them on solid media at 32°C. These plates demonstrate that most (∼80%) of the *mph1nda3* double mutants lose viability in their first division at 18°C (Figure 1D). Even when maintained at their permissive temperature (30°C), there are significant numbers of inviable cells (defined as those unable to form a colony) in the *mph1nda3* double-mutant population, reflecting inherent chromosome loss and aneuploidy in these *mph1* alleles. These experiments demonstrate the importance of Mph1 kinase signaling in fission yeast mitosis and are consistent with vertebrate studies employing chemical inhibitors of Mps1 [8].

### *mph1* Mutants Display Reduced Mad2 and Mad3-APC/C Binding

**Figure 2:**
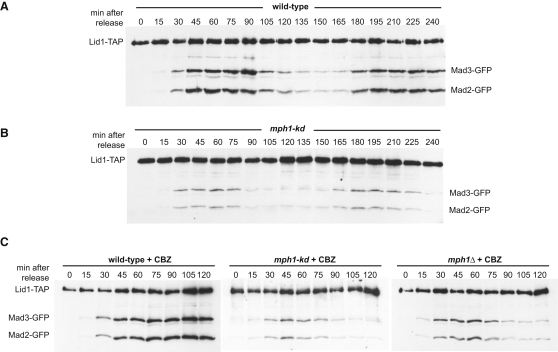
*mph1* Mutants Display Reduced MCC-APC/C Binding (A and B) Strains containing Lid1-TAP, Mad2-GFP, and Mad3-GFP were prearrested in G2 with the *cdc25 ts* mutation, before releasing for two cell cycles. Extracts were made from each time point the APC/C was pulled down, washed, and immunoblotted for associated Mad2- and Mad3-GFP. (C) Similar binding assays were performed but an antimicrotubule drug, 50 μg/ml CBZ, was added at 20 min after *cdc25* release.

Fission yeast Mad2p and Mad3p bind to the APC/C in mitosis [15], and we employed *cdc25* block and release to determine whether this interaction was perturbed in the absence of Mph1 kinase activity. *cdc25* cells containing Lid1(Apc4)-TAP, Mad2-GFP, and Mad3-GFP were arrested in G2 at 36°C and then released, to pass through a synchronous mitosis at 25°C. Samples were taken at 15 min time points, native extracts made, and Lid1-associated complexes were analyzed by pull-down and immunoblotting (Figure 2A). Mad2p and Mad3p displayed a significantly reduced ability to bind stably to fission yeast APC/C in *mph1-kd* (Figure 2B). Thus, Mph1 kinase is required for stable association of fission yeast spindle checkpoint proteins with mitotic APC/C. Another important observation can be made: in this experiment two cell cycles occurred and two cycles of APC/C binding were apparent. Thus Mad2p and Mad3p bind to the APC/C each mitosis, even under optimal growth conditions, demonstrating that the fission yeast spindle checkpoint is active every cell cycle.

We repeated this experiment adding an antimicrotubule drug (50 μg/ml carbendazim, CBZ) 20 min after release from the *cdc25* (G2) block. Such timing enabled cells to enter mitosis before spindle microtubules were depolymerized. In Figure 2C we compared wild-type, *mph1Δ*, and *mph1-kd* strains for their ability to form MCC-APC/C complexes and checkpoint arrest. Both *mph1* mutants failed to assemble significant levels of MCC-APC/C, and both failed to checkpoint arrest, highlighting the importance of Mph1 kinase activity in the spindle checkpoint. We conclude that Mph1 kinase activity is required to assemble and/or maintain MCC-APC/C complexes.

### Mad2p Phosphorylation Site Mutants Are Checkpoint Defective

**Figure 3:**
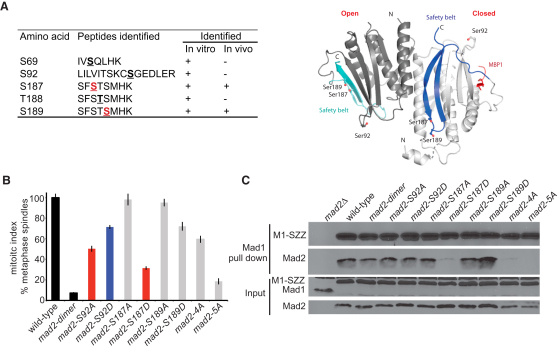
*mad2* Phospho Mutants Can't Maintain Checkpoint Arrest (A) Summary of the phospho-peptides identified after mass-spectrometric analyses of Mad2p. This model of an *S. pombe* Mad2 dimer is based on PDB ID 2V64, showing the likely positions of serine residues that are phosphorylated. The model was generated by applying the *S. pombe* Mad2 sequence (44% identity) to the human MAD2L1 crystal structure with the program CHAINSAW from the CCP4 crystallographic software suite [30]. (B) Certain *mad2* alleles fail to maintain *nuf2-3* spindle checkpoint arrests. *nuf2-3* strains were constructed containing the indicated *mad2* alleles, grown to log phase at 25°C, and then shifted to 32°C for 4 hr. Cells were then fixed and spindles stained with tubulin antibodies. This experiment was repeated at least three times and error bars indicate the standard deviation. (C) Single serine substitutions in Mad2p are stable and, with the notable exception of S187D, bind Mad1p efficiently. Whole-cell extracts were made and Mad1-SZZ pulled down with IgG Dynabeads. Extracts (input) and pull downs were immunoblotted for Mad1-SZZ and Mad2p. The *mad2-4A* and *5A* substitutions (*mad2-4A* [S69A, S92A, S187A, S189A] and *mad2-5A* [S69A, S92A, S187A, T188A, S189A]) express unstable proteins (60% and 34%, respectively, when compared to wild-type Mad2p). See also Figures S2–S4.

Inhibition of Mps1 kinase in vertebrate cells leads to the depletion of Mad2 from kinetochores and perturbs the regulation of mitotic timing [8]. Mad2 needs to dimerize, and it is possible that it needs to be phosphorylated, before it is converted to a conformation that stably binds and inhibits Cdc20-APC/C. In a recent study it was argued that sustained Mps1 activity is required to recruit Mad2 to the kinetochore-localized Mad1-Mad2 template [16]. Although budding yeast Mad1 appears to be a good Mps1 kinase substrate [17], preliminary experiments in fission yeast failed to demonstrate clear Mad1 phosphorylation. Therefore, we tested whether fission yeast Mad2p is an Mph1 substrate. First we carried out mass-spectrometric analyses of in vitro Mph1 kinase assays with Mad2p substrate (Figure 1A). This analysis was performed both with and without phosphopeptide enrichment on titanium oxide beads (see Experimental Procedures) and we identified 5 in vitro Mph1 sites in Mad2p and 15 on Mph1 kinase, 2 of which (T470 and T471) are in its predicted activation segment (Figure S2). To confirm the Mad2p phosphorylation sites in vivo, we performed tandem affinity purification of Mad2-His-TEV-Biotin (HTB) from both cycling and *nda3* arrested cells. The HTB tag enables purification of Mad2p from denatured fission yeast extracts, in which protein phosphatases are inactive. Two of the in vitro sites (S187 and S189) were confirmed in vivo and are summarized in Figure 3A (see Figures S4 and S2 for Mad2 phospho-peptide spectra, alignments, and Mph1 sites). Phospho-specific Mad2 antibodies were generated (Figure S3), but unfortunately these lack the sensitivity needed to detect modified forms of Mad2p in complexes purified from yeast.

To test whether modification of these residues in Mad2p is important for checkpoint arrest, we made mutations, alone and in various combinations, substituting serine and threonine residues with nonphosphorylatable alanine (A) or phospho-mimicking aspartic acid (D). Mad2 dimerization is known to be critical for checkpoint signaling, and we made an R133A, Q134A double substitution in fission yeast Mad2p as a *mad2* loss-of-function control allele for these experiments. We tested the *mad2* alleles for their ability to arrest cells containing kinetochore defects, by using the temperature-sensitive *nuf2-3* allele at 32°C where ∼30% of cells arrest with short spindles after 4 hr (Figure 3B). Although the *mad2* phospho mutants weren't as defective as the *mad2* dimerization mutant, this experiment directly demonstrates that mutation of Mph1 phosphorylation sites in Mad2p (S92 and S187) significantly reduces a cell's ability to checkpoint arrest.

### Mad2p Modification Can Disrupt Mad1p-Mad2p Complexes

Single *mad2-SA* mutations didn't significantly impair Mad2p stability or Mad1p binding (Figure 3C), demonstrating that none of these phospho-serine modifications are critical for this interaction. The *mad2-4A* (S69A, S92A, S187A, S189A) and *mad2-5A* (S69A, S92A, S187A, T188A, S189A) mutants displayed significantly reduced Mad1p binding, but these mutants expressed less stable forms of Mad2p (Figure 3C). Because reduced binding between Mad2p and Mad1p is not observed in an *mph1-kd* allele (not shown), we believe that these mutations are likely to have perturbed Mad2p structurally and that their phenotypes shouldn't be interpreted as being due to lack of posttranslational modification.

Previous work argued that phospho-mimic substitutions in C-terminal residues of human Mad2 can inhibit both Mad1 and Cdc20-APC/C binding [18], perhaps by negatively regulating Mad2 conformational transitions [19]. The human Mad2 phospho-sites are not conserved in yeast Mad2p, but one of the phospho-mimic mutations in the C terminus of fission yeast Mad2p did display a similar phenotype. *mad2-S187A* had no phenotype, demonstrating that phosphorylation of S187 isn't necessary for checkpoint arrest and probably doesn't contribute to the requirement for Mph1 activity in spindle checkpoint arrest. However, the phospho-mimic *mad2-S187D* allele displayed significantly reduced Mad1p binding (Figure 3C). *mad2-S189D* (just two residues away) had no such defect, highlighting the specificity of the S187 modification and apparently ruling out any simple explanation resulting from addition of negative charge within the C-terminal Mad2p safety belt. *mad2-S187N* also has no phenotype, confirming that it is the additional charge that is disruptive in *S187D* (Figure S3B). We don't know whether it is the open or closed conformation of Mad2p that is phosphorylated, but one interpretation of the *mad2-S187D* allele is that modification of Mad2p by Mph1 kinase could disrupt Mad1p-Mad2p complexes (see Figure S3A for structural models). This phenotype is consistent with the human Mad2 phosphorylation studies [18] and suggests that Mph1 kinase activity could have inhibitory checkpoint signaling roles, perhaps disrupting Mad1-Mad2 kinetochore scaffolds during checkpoint silencing and/or interphase. Such a negative role would be an entirely novel function for Mph1 kinase. However, we can't rule out that S187 is phosphorylated by additional protein kinases in vivo; indeed, it could be an unidentified kinase that negatively regulates the Mad1-Mad2 complex. Whatever the kinase(s) responsible, these C-terminal residues are very well conserved (SFSTS is SFTTT in human and TFSTN in *S. cerevisiae*; see Figure S2), and when we made the equivalent substitution in *S. cerevisiae* Mad2p (S182D), we observed similar checkpoint defects indicating that negative regulation may also be conserved.

### Mad2p Phosphorylation Is Important for Mad2 and Mad3-APC/C Binding

The reduced ability of *mad2-S187D* to bind Mad1p can explain its compromised checkpoint arrest, but why do S92 substitutions perturb checkpoint arrest?

**Figure 4:**
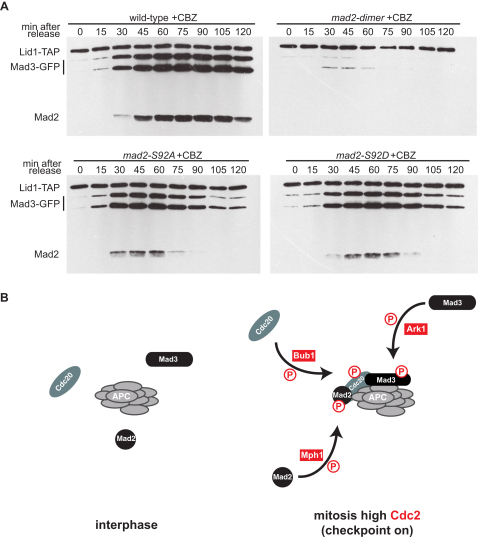
*mad2-S92* Alleles and Nondimerizing Mutants Display Reduced Cdc20^Slp1^-APC/C Binding (A) Strains expressing the indicated *mad2* alleles (not tagged), Lid1-TAP, and Mad3-GFP were prearrested in G2 with the *cdc25 ts* mutation, before releasing into mitosis and adding CBZ. Extracts were made from each time point, and the APC/C pulled down, washed, and immunoblotted for associated Mad2p and Mad3-GFP. (B) Combinatorial phosphorylation model: combined actions of CDK, Mph1, Ark1, and Bub1 kinases lead to the stabilization of MCC-APC/C complexes in mitosis. Their actions can be reversed by PP1^Dis2^ upon satisfaction of the spindle checkpoint [26].

To test whether mutation of S92 perturbed Cdc20^Slp1^-APC/C binding, we carried out *cdc25* (G2) block and release time courses and immunoprecipitated the APC/C from *mad2-92A* and *mad2-92D* mutants at each time point (Figure 4A). CBZ was added 20 min after *cdc25* release to test whether *mad2* alleles could maintain checkpoint arrest. We conclude that Mad2p dimerization and phosphorylation are both important for stable binding of Mad2p and Mad3p to APC/C. Both S92A and S92D have significantly reduced interaction with Cdc20^Slp1^-APC/C, although S92D is more competent than S92A (reproducibly displaying APC/C binding up to 75 min in the time course, rather than 60 min). We conclude that S92 phosphorylation is important for stable Cdc20^Slp1^-APC/C interactions and in particular for maintenance of checkpoint arrest. *mad2-S92A* is a “separation of function” allele: although *mph1-kd* and *mad2Δ* have defects in both checkpoint activation and maintenance, *mad2-S92A* is specifically defective in maintenance.

We propose that phosphorylation of Mad2 by Mph1 kinase is one of several posttranslational modifications of the MCC (Mad2-Mad3-Cdc20^Slp1^) that stabilize the MCC-APC/C interaction and thereby inhibit APC/C activity during checkpoint arrest. Reduced MCC-APC/C interaction could result from reduced upstream signaling functions such as Ndc80 phosphorylation [20], but it is likely that Mph1 kinase also acts directly on MCC components [21]. *mad2-SA* alleles have weaker phenotypes than *mph1-kd*, suggesting that additional Mph1 substrates remain to be identified. Likely candidates include Mad3, Cdc20^Slp1^, and APC/C subunits. Aurora kinase (Ipl1 in budding yeast) phosphorylates ScMad3 [22] and Bub1 kinase phosphorylates human Cdc20 [23, 24]. We have identified 16 in vitro Mph1 sites in Mad3p and propose that they act in combination with the Mad2p modifications described here to stabilize MCC-APC/C (Figure 4B).

### Concerted Actions of Aurora and Mph1 Kinases

Vertebrate Aurora B acts early in mitosis to recruit Mps1 to kinetochores, to potentiate its activity, and to establish checkpoint arrest [25]. We propose that Mph1 and Ark1 kinases are key regulators of checkpoint arrest in fission yeast, with important roles at kinetochores in initiating checkpoint signals, and also in the nucleoplasm where they directly regulate MCC and MCC-APC/C stability [21, 26] to maintain checkpoint arrest. Precisely how these kinases are regulated, and how their actions are then opposed by phosphatases such as PP1^Dis2^ to drive anaphase onset, are important questions for the future [27]. Recent experiments identified Spc7/Spc105/KNL1/Blinkin and the kinesins Klp5/6 as key recruitment factors for PP1 at kinetochores [28, 29], but how PP1 recruitment and activity is regulated remains to be determined. No doubt both Mps1 and Aurora B kinases will have something to say in this regard.

## Supplemental Information

Document S1. Supplemental Discussion, Supplemental Experimental Procedures, Figures S1–S4, and Table S1
