# Supplementary material for: Kinase Activity of Fission Yeast Mph1 Is Required for Mad2 and Mad3 to Stably Bind the Anaphase Promoting Complex
Source: Curr Biol. 2012 Feb 21;22(4):296–301. doi: 10.1016/j.cub.2011.12.049 (PMC3315010; doi:10.1016/j.cub.2011.12.049)
Supplement: Document S1. Supplemental Discussion, Supplemental Experimental Procedures, Figures S1–S4, and Table S1 [file mmc1.pdf]

Current Biology, Volume *22*

Supplemental Information

Kinase Activity of Fission Yeast Mph1 Is Required for Mad2 and Mad3 to Stably Bind the Anaphase Promoting Complex

Judith Zich, Alicja M. Sochaj, Heather M. Syred, Laura Milne, Atlanta G. Cook, Hiro Ohkura, Juri Rappsilber, and Kevin G. Hardwick

Inventory of Supplementary Data:

Figure S1 (related to Figure 1)

Figures S2-S4 (related to Figure 3)

Table S1. Yeast strains

Supplemental Discussion

Supplemental Experimental Procedures

Supplemental References

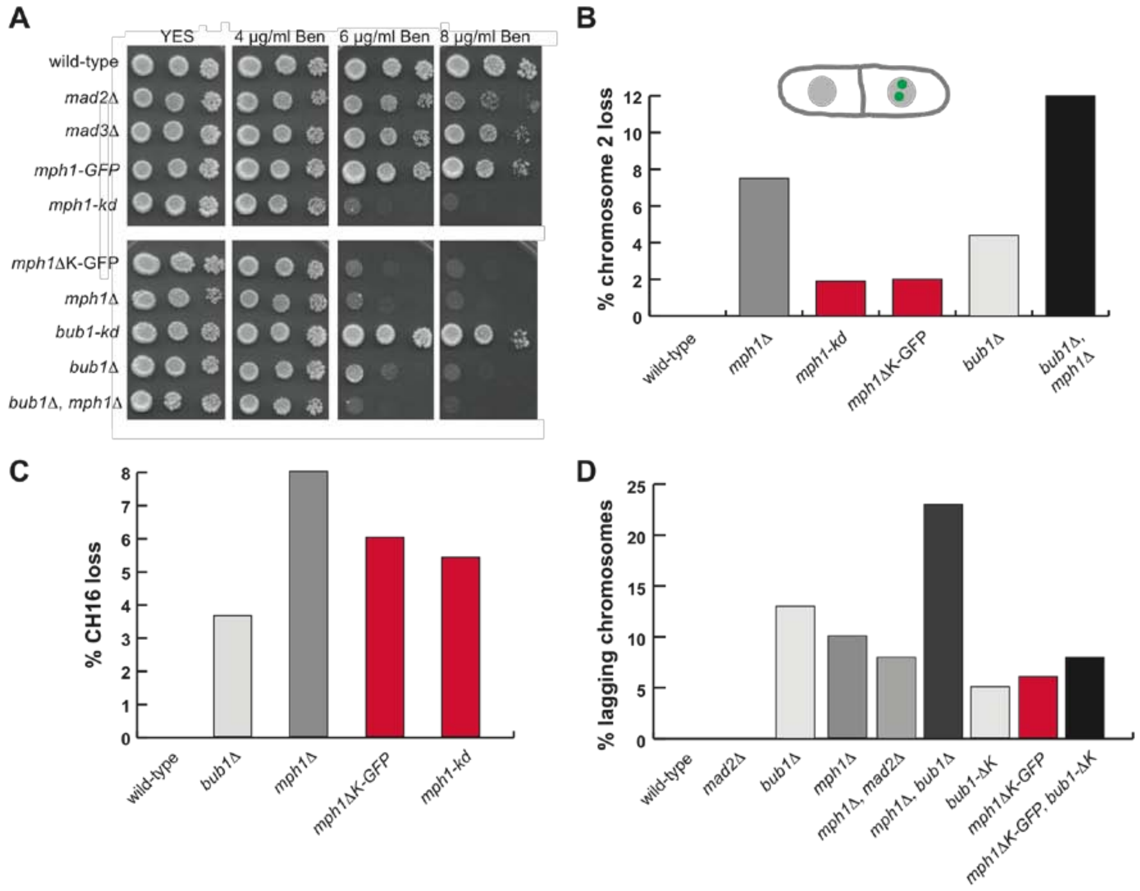

Figure S1. Benomyl sensitivity and chromosome loss phenotypes of *mph1* kinase dead mutants.
A) *mph1* kinase dead alleles are benomyl (anti-microtubule drug) sensitive. The indicated strains were diluted, plated on rich media (YES) with or without benomyl and then grown for 3 days at 30°C.
B) *mph1* kinase dead alleles mis-segregate chromosome 2. The indicated strains containing cen2-GFP were grown in liquid cultures and then bi-nucleate cells carefully analysed and scored for segregation of chromosome 2. N was >900 cells for all strains, with the experiment repeated at least 3 times for each strain.
C) The indicated strains, containing the Ch16 mini-chromosome, were plated on low adenine containing media and colonies analysed for sectoring. Loss of Ch16 in the first division results in a half-sectored colony (half pink, half white). Total colony number was >3000 for all strains, and is the combination of at least 3 experiments for each strain.
D) mph1 kinase dead alleles display lagging chromosomes. Strains were grown, fixed and analysed by tubulin immunofluorescence and DAPI staining. The percentage of anaphase cells containing lagging chromosomes were scored (these are frequently merotelically attached single sisters in fission yeast [1]).
N of cells was >1000 for each strain, and was repeated at least 3 times for each strain. These data are also summarised in Fig1B.

Figure S1 and Fig 1B demonstrate significant chromosome loss rates for the *mph1-kd* alleles, for example they are far higher than *mad2*Δ or *mad3*Δ strains. The loss rates are even higher for the complete deletion, which argues that the N-terminal domains of this protein are likely to have additional, kinase-independent functions.
These data also demonstrate that the double mutants, *bub1*Δ *mph1*Δ, and *bub1-ΔK mph1ΔK*, have higher rates of chromosome loss than single mutants. Such synthetic phenotypes can be

interpreted in various ways: (i) the two protein kinases may share substrates (in this scenario, absence of post-translational modification on both Mph1 and Bub1 sites in “substrate X” leads to a more severe loss of function than loss of solely Mph1-dependent or Bub1-dependent modification); (ii) the kinases may share some but not all substrates, or (iii) the kinases have distinct targets thereby affecting multiple pathways. Unfortunately the only way to distinguish between these possibilities is to identify their respective kinetochore targets, and this has yet to be done for Mph1 kinase. We conclude that Mph1 and Bub1 both have roles in chromosome segregation that are distinct from spindle checkpoint arrest and to an extent distinct from one another. This is supported by known genetic interactions: whilst many genetic interactions are shared there are some differences, for example *bub1*Δ is synthetic lethal with *klp5*Δ but this is not true for *mph1*Δ [2]. We have confirmed this observation and were unable to construct an *nda3, mph1*Δ*, bub1*Δ triple mutant strain (LM, data not shown) again suggesting non-redundant segregation functions for these two protein kinases.

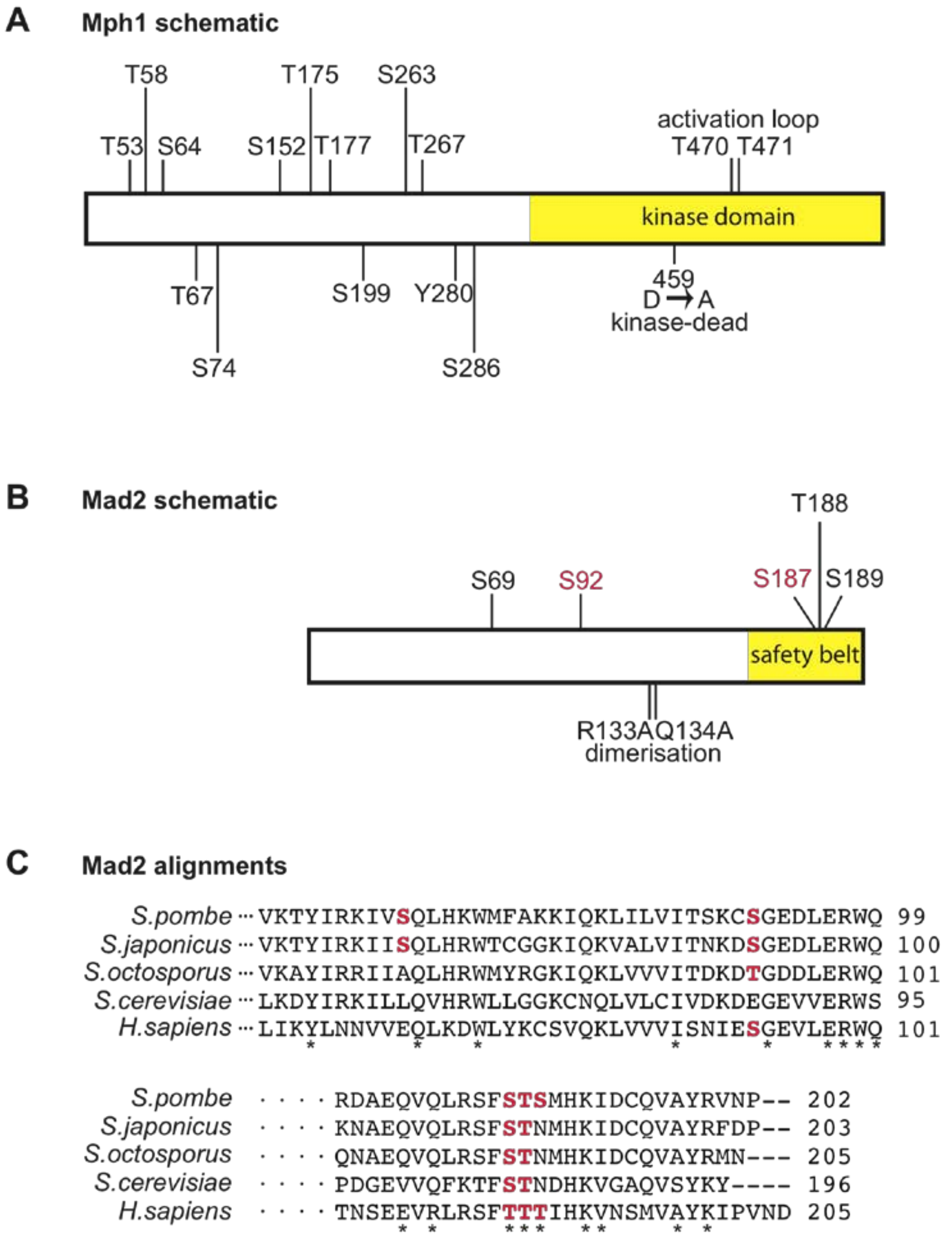

Figure S2 (related to main text Fig 3). Mad2 and Mph1 phosphorylation site schematics and sequence alignments.

A) Mph1 schematic indicating the auto-phosphorylation sites identified by mass spectrometry from the *in vitro* Mph1 kinase assay. The kinase-dead point mutation is also indicated.

B) Mad2 schematic indicating the five *in vitro* phosphorylation sites (the two confirmed in vivo are labelled in red). The dimerisation mutant is also shown.

C) Mad2 sequence alignments with conserved phospho-sites indicated in red.

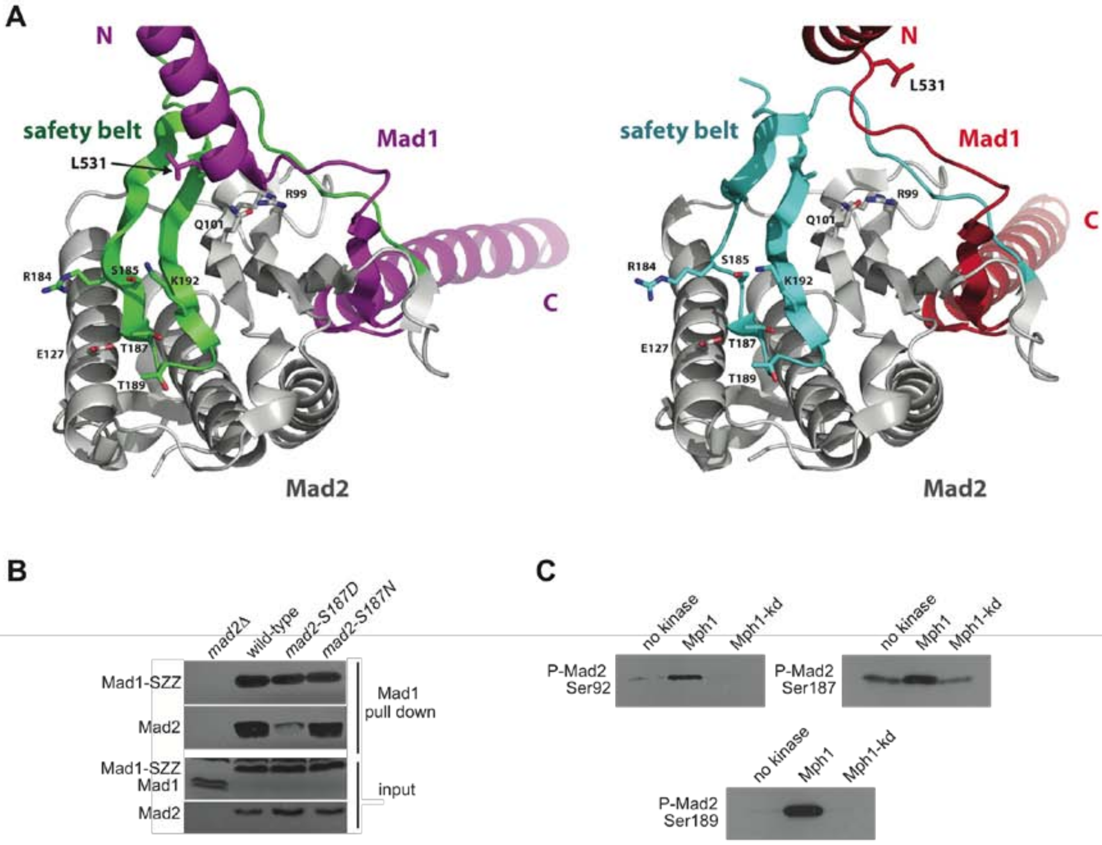

Figure S3 (related to main text Fig 3). *mad2-S187D* displays reduced Mad1 binding.

A) **Structural models explaining the importance of S187 modification.** See supplementary discussion for further details and explanation of these models.

B) **Mad1 pull downs from *mad2-S187D* and *mad2–S187N* alleles.**
Whilst an aspartic acid substitution at position 187 disrupts the Mad2-Mad1 interaction, this is not the case for an asparagine substitution, indicating that it is the charge, rather than the size, that is disruptive.

C) **The phospho-specific anti-Mad2 antibodies** confirm that serines 92, 187 and 189 can all be modified by Mph1 kinase *in vitro*. Unfortunately they are not sensitive enough to detect modifications on Mad2p purified from fission yeast cells.

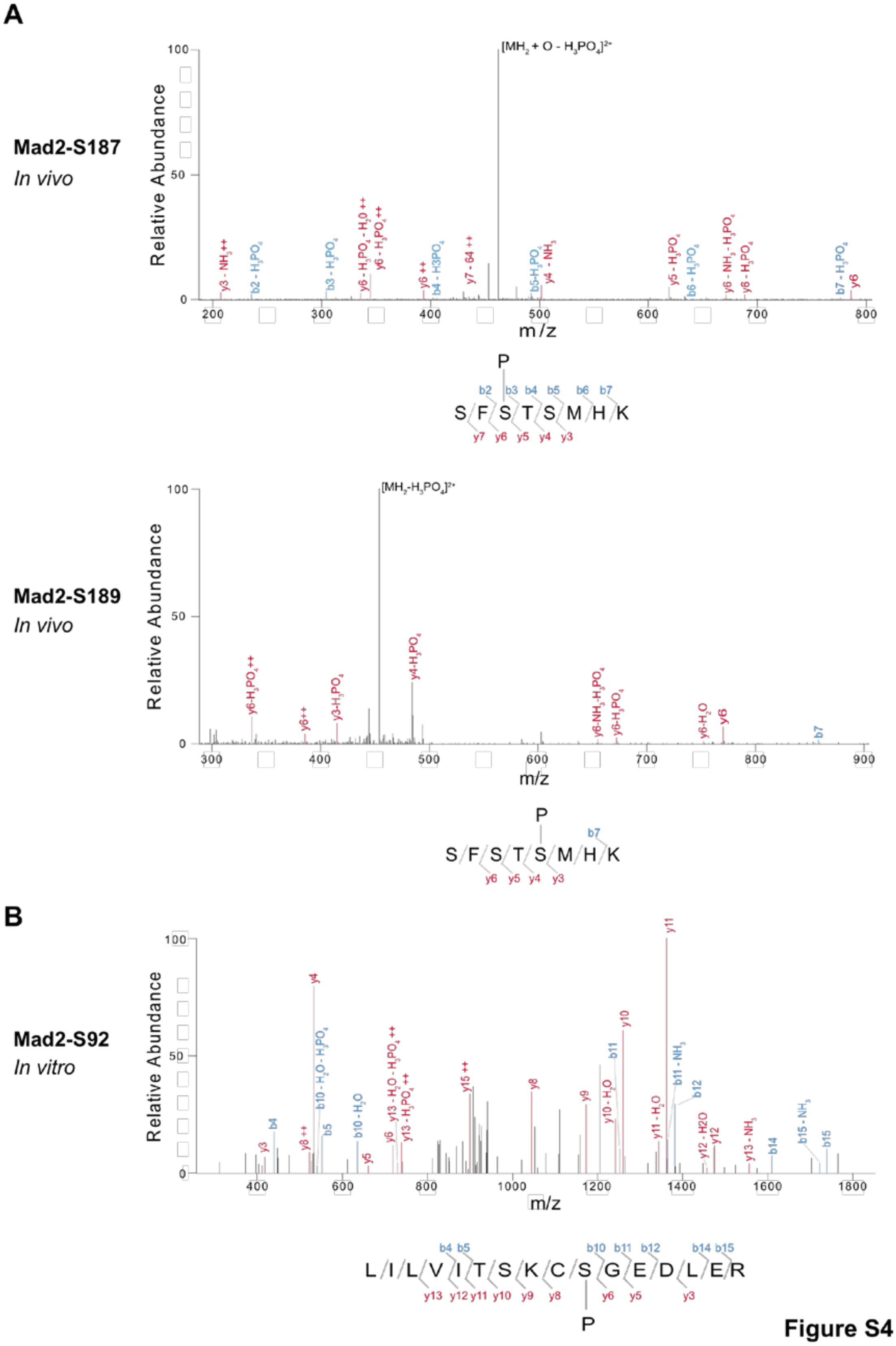
A
Mad2-S187
In vivo
Relative Abundance
m/z
[MH2 + O - H3PO4]2+
S F S T S M H K
P
Mad2-S189
In vivo
Relative Abundance
m/z
[MH2-H3PO4]2+
S F S T S M H K
P
B
Mad2-S92
In vitro
Relative Abundance
m/z
L I L V I T S K C S G E D L E R
P

Figure S4

Figure S4 (related to main text Fig 3). MS/MS spectra from mass spectrometric analyses of Mad2p.

Examples of MS/MS spectra used to identify and assign phosphorylation of each phosphopeptide identified. Fragment ions containing the peptide's N- (b-ions) or C- (y-ions) termini are labelled. A number of the identified peptides show neutral loss of phosphoric acid from the full length peptide, supporting the conclusion of peptide phosphorylation. Significant fragmentation has also occurred allowing peptide sequencing and assignment of the phosphorylated residue.

A) Examples of spectra for the Mad2 phosphorylation sites identified from Mad2-HTB purified from *nda3* arrested and/or cycling fission yeast cells (*in vivo*).

B) One example (S92) spectrum for Mad2 phosphorylation sites identified from *in vitro* Mph1 kinase assays. Spectra for other sites (S69, S187, T188, S189) are available upon request.

Supplemental Discussion

A possible structural explanation for the effects of S187D mutation

The 2:2 tetrameric Mad1-Mad2 complex is significantly asymmetric, with different orientations of Mad1 with respect to Mad2 (see Figure S3). The structure of two Mad1-Mad2 dimers that make up the human complex are shown in the same orientation with respect to Mad2 (PDB id 1GO4, [3]). Several residues that are identical in human and *S.pombe* are shown as sticks. The left dimer shows a compact association with the N-terminal helix of Mad1. This interaction is mediated by the burial of Leu531Mad1 in a hydrophobic pocket on the last two β-strands of Mad2. The interaction is further stabilised by the packing of the N-terminal helix of Mad1 against residues Arg99Mad2 and Gln101Mad2. The right dimer shows looser packing, where the end of the N-terminal helix is partially melted and the close contact with Leu531Mad1 is lost. In this conformation the integrity of the 8th β-strand is also disrupted. This asymmetry has been hypothesized to be functionally significant as it may provide a "fracture line" or weak point where Mad1-Mad2 complexes could be disassembled [3].

Thr187 and Thr189, the human equivalents of S187 and S189 in *S.pombe*, lie at the end of the 8th β-strand of Mad2. Thr187 lies at the end of the zone that is disrupted in the loose-packed conformation and close to Glu127, a conserved residue on a neighbouring helix. Mutation of this threonine residue to aspartic acid could lead to an unfavourable clash with Glu127 that might disrupt the β-strand. This could, in turn, favour the looser conformation (seen on the right) over the more compact interaction (left), thus destabilising the interaction between Mad1 and Mad2. Figures were produced using Pymol (The PyMOL Molecular Graphics System, 2006 Delano Scientific LLC).

Table S1. Fission yeast strains

**Figure1**	KP114	wild-type: *ura4-D18 leu1-32 ade-210*	This lab
	YJZ24	*mph1-kd-SZZ:leu1*$^+$ *ura4-D18 leu1-32 ade-210*	This lab
	YJZ4	*mph1SZZ:leu1*$^+$ *ura4-D18 leu1-32 ade-210*	Allshire
	YJZ67	*mph1-kd:leu1*$^+$ *ura4-D18 ade-210*	This lab
	YLM60	*mph1Δ::NAT leu1-32 ade6-210 ura4-D18 h*$^-$	This lab
	KP135	*mad3Δ::ura4*$^+$*, ade6-210, leu1-32, ura4-D18 h*$^-$	This lab
	YLM109	*mph1-GFP:kan*^{r} *ade6-210 leu1-32 ura4-D18 his3-D1 arg3-D4 h*$^-$	This lab
	YLM111	*mph1KΔ-GFP:kan*^{r} *ade6-210 leu1-32 ura4-D18 his3-D1 arg3-D4 h*$^-$	This lab
	KP260	*bub1-K762M*	Javerzat
	KP064	*bub1Δ::ura4*$^+$	This lab
	YLM62	*mph1Δ::NAT bub1Δ::ura4*$^+$ *leu1-32 ade6-201 ura4-D18 h*$^+$	This lab
	KP349	*cen2D107(::Kan-ura4-lacO) his7::lacI-GFP leu1-32 ade36-M210 lys1 ura4*	Hiraoka
	YJZ171	*cen2D107(::Kan-ura4-lacO) his7::lacI-GFP mph1-kd:leu1*$^+$	This lab
	YLM42	*cen2D107(::Kan-ura4-lacO) his7::lacI-GFP mph1Δ::NAT ade6-210 ura4-D18 lys1*	This lab
	YLM135	*cen2D107(::Kan-ura4-lacO) his7::lacI-GFP mph1KΔ-GFP:kan*^{r} *ade6-210 ura4-D18 lys1*	This lab
	YLM43	*cen2D107(::Kan-ura4-lacO) his7::lacI-GFP bub1Δ::ura4*$^+$ *ade6-210 ura4-D18 lys1*	This lab
	YLM63	*cen2D107(::Kan-ura4-lacO) his7::lacI-GFP mph1Δ::NAT bub1Δ::ura4*$^+$ *leu1-32 ade6-201*	This lab
	JPJ499	*CH16 (bub1Δ::ura4*$^+$*)*	Javerzat
	YJZ193	*CH16 (bub1Δ::ura4*$^+$*) mph1-kd:leu1*$^+$	This lab
	JPJ502	*CH16 (bub1Δ::ura4*$^+$*) bub1Δ::LEU2*$^+$	Javerzat
	YLM22	*CH16 (bub1Δ::ura4*$^+$*) mph1Δ::ura4*$^+$	This lab
	YLM190	*CH16 (bub1Δ::ura4*$^+$*) mph1ΔK-GFP:kan*^{r}	This lab
	YLM202	*nda3-KM311 Plo1-GFP:ura4*$^+$ *h*$^-$	Okhura
	YJZ99	*nda3-KM311 Plo1-GFP:ura4*$^+$ *mph1-kd:leu1*$^+$	This lab
	YJZ128	*nda3-KM311 Plo1-GFP:ura4*$^+$ *mad2Δ::ura4*$^+$	This lab
	YLM201	*nda3-KM311 Plo1-GFP:ura4*$^+$ *mph1Δ::NAT leu1-32 ade6-210 ura4-D18*	This lab
	KP340	*nda3-KM311 h*$^-$	This lab
	YJB245	*nda3-KM311 mad2Δ::ura4*$^+$	This lab
	YJZ73	*nda3-KM311 mph1-kd:leu1*$^+$	This lab
	YLM83	*nda3-KM311 mph1Δ::NAT*	This lab
**Figure 2**	KP434	*cdc25-22 lid1-TAP:kan*^{r} *mad3-GFP::his3*$^+$ *mad2-GFP::his3*$^+$	This lab
	YJZ162	*cdc25-22 lid1-TAP:kan*^{r} *mad3-GFP::his3*$^+$ *mad2-GFP::his3*$^+$ *mph1-kd:leu1*$^+$	This lab
	KP443	*cdc25-22 lid1-TAP:kan*^{r} *mad3-GFP::his3*$^+$ *mad2-GFP::his3*$^+$ *mphΔ::NAT*	This lab
**Figure 3**	YJZ311	*nuf2-3:ura4*$^+$ *mad2:leu1*$^+$	This lab
	YJZ295	*nuf2-3:ura4*$^+$ *mad2-dimer:leu1*$^+$	This lab
	YJZ290	*nuf2-3:ura4*$^+$ *mad2-S92A:leu1*$^+$	This lab
	YJZ294	*nuf2-3:ura4*$^+$ *mad2-S92D:leu1*$^+$	This lab
	YJZ327	*nuf2-3:ura4*$^+$ *mad2-S187A:leu1*$^+$	This lab
	YJZ324	*nuf2-3:ura4*$^+$ *mad2-S187D:leu1*$^+$	This lab
	YJZ326	*nuf2-3:ura4*$^+$ *mad2-S189A:leu1*$^+$	This lab
	YJZ325	*nuf2-3:ura4*$^+$ *mad2-S189D:leu1*$^+$	This lab
	YJZ292	*nuf2-3:ura4*$^+$ *mad2-S69AS92AS187AS189A:leu1*$^+$	This lab
	YJZ293	*nuf2-3:ura4*$^+$ *mad2-S69AS92AS187AT188AS189A:leu1*$^+$	This lab
	YJZ312	*mad1-TAP:kan*^{r} *mad2:leu1*$^+$	This lab
	YJZ310	*mad1-TAP:kan*^{r} *mad2-dimer:leu1*$^+$	This lab
	YJZ305	*mad1-TAP:kan*^{r} *mad2-S92A:leu1*$^+$	This lab
	YJZ309	*mad1-TAP:kan*^{r} *mad2-S92D:leu1*$^+$	This lab

	YJZ331	*mad1-TAP:kan*^{r} *mad2-S187A:leu1*$^+$	This lab
	YJZ328	*mad1-TAP:kan*^{r} *mad2-S187D:leu1*$^+$	This lab
	YJZ330	*mad1-TAP:kan*^{r} *mad2S189A:leu1*$^+$	This lab
	YJZ329	*mad1-TAP:kan*^{r} *mad2-S189D:leu1*$^+$	This lab
	YJZ328	*mad1-TAP:kan*^{r} *mad2-S187N:leu1*	This lab
	YJZ307	*mad1-TAP:kan*^{r} *mad2-S69AS92AS187AS189A:leu1*$^+$	This lab
	YJZ308	*mad1-TAP:kan*^{r} *mad2-S69AS92AS187AT188AS189A:leu1*$^+$	This lab
	AE148	*mad2Δ::ura4*$^+$ *h*$^-$	Matsumoto
	AS031	*mad2-HTB:kan*^{r} *nda3-KM311*	This lab
**Figure 4**	YJZ313	*cdc25-22 lid1-TAP:kan*^{r} *mad3-GFP:his3*$^+$ *mad2:leu1*$^+$	This lab
	YJZ318	*cdc25-22 lid1-TAP:kan*^{r} *mad3-GFP:his3*$^+$ *mad2-dimer:leu1*$^+$	This lab
	YJZ314	*cdc25-22 lid1-TAP:kan*^{r} *mad3-GFP:his3*$^+$ *mad2-S92A:leu1*$^+$	This lab
	YJZ317	*cdc25-22 lid1-TAP:kan*^{r} *mad3-GFP:his3*$^+$ *mad2-S92D:leu1*$^+$	This lab

Supplemental Experimental Procedures:

Generation of Mad2 phosphorylation mutants

Phosphorylation mutants were created from pJK-Mad2, which is based on the vector pJK148 [4]. It contains 250 bp of 5'UTR, followed by the *mad2* ORF and 150 bp of 3'UTR. All *mad2* strains were generated by integration of pJK-Mad2 vectors into a *mad2Δ* strain at the endogenous *mad2* locus. Point mutations were introduced to replace S69, S92, S187, T188, S189 with alanine or aspartic acid using the Quikchange Kit for Site Directed Mutagenesis (Stratagene).

Generation of *mph1-kd* and *mph1-kd-SZZ*

The *mph1-kd* strain was generated by integration of pJK *mph1-kd* into a wild-type *mph1* strain at the endogenous *mph1* locus. pJK *mph1-kd* is based on the vector pJK148 [4]. It contains the *mph1* sequence from base 408 to the end of *mph1* followed by ~200bp of 3'UTR. The kinase-dead mutation was generated by introducing a single point-mutation at base A1376C. The *mph1-kd-SZZ* strain was generated similar to *mph1-kd* with the addition of the insertion of a C-terminal SZZ prior to the 3'UTR.

Mitotic arrests

Yeasts strains are listed in Table 1. *nda3-KM311*(cs) cells were grown overnight in yeast extract plus supplements (YES) medium at 30°C to mid-log phase and then shifted to 18°C for 6 h. For microscopy cells were briefly fixed at -20°C in 100 % methanol and mounted with 1 mg/ml DAPI (Sigma). For viability assays cells were arrested at 18°C for 6 h and single cells plated on YES plates and incubated at 32°C. For *nuf2-3* arrests cells were grown overnight in YES medium at 25°C to mid-log phase and then shifted to 32°C for 4 h followed by fixation in -20°C 100 % methanol.

G2 arrests (*cdc25-22*)

For *cdc25-22* arrests cells were grown overnight in YES medium at 25°C to mid-log phase and then shifted to 36°C for 3.5 h. To release cells from the G2 arrest cultures were cooled down rapidly to 25°C and then incubated at 25°C.

Benomyl sensitivity assay

10 fold serial dilutions were plated on YES plates containing 0, 4, 6, 8 μg/ml of the microtubule depolymerising drug benomyl. Plates were incubated at 30°C for 3 days.

Chromosome mis-segregation assays

Cen2-gfp: Overnight cultures were grown to mid-log phase in YES and the rate of cen2-gfp mis-segregation scored in binucleate live cells.

CH16 minichromosome loss: The CH16 strain used in this assay contains a *ura4* marked *bub1*Δ [5]. Overnight cultures were grown to mid-log phase in PMG lacking uracil and adenine. 250 cells were plated on YES plates containing low adenine (6.6 µg/ml). Plates were incubated for 4 days at 30°C and the percentage of CH16 mini-chromosome loss scored as a percentage of half-sectored colonies to white colonies. For more details on chromosome segregation assays see [5].

Immunostaining

10–50 ml of overnight culture were harvested and then fixed with -80°C cold methanol and washed twice with PEM (100 mM PIPES, pH 7.6, 1 mM $MgSO_4$, 1 mM EGTA). Cell walls were then digested in PEMS (100 mM PIPES, pH7.6, 1 mM $MgSO_4$, 1 mM EGTA, 1 M sorbitol) with 0.4 mg/ml Zymolyase (MP Bio-medicals) for 30-45 min. Cells were then washed once with PEMS, PEMS-TritonX-100 (1 %) and PEM. Cells were blocked with PEMBAL (1 % BSA, 0.1 % L-Lysine in PEM) for 1 h and then incubated overnight with TAT1 (mouse anti-tubulin) antibody (1:50) (kindly provided by Keith Gull, Oxford, UK). Cells were washed once with PEM and then incubated with anti- mouse secondary antibody (Alexa Fluor – Molecular Probes) at 1:1000 for 1 h. Mitotic spindles were visualized using an Intelligent Imaging Innovations Marianas microscope (Zeiss Axiovert 200M, using a x100 1.3NA objective lens), CoolSnap CCD, and Slidebook software (Intelligent Imaging Innovations, Inc., Boulder, CO).

Immunoprecipitations

Anaphase promoting complex interaction

Cells expressing TAP-tagged Lid1 (Apc4) (original strain kindly provided by Kathy Gould, Vanderbilt, USA) and Mad2p and Mad3p tagged with GFP from their endogenous loci were presynchronized in G2 via the *cdc25-22* mutation. Proteins were extracted in lysis buffer (50 mM HEPES [pH 7.5], 75 mM KCl, 1 mM $MgCl_2$, 1 mM EGTA, 0.1% TritonX-100, 1 mM sodium vanadate, 0.1 µM microcystin, 10 µg/ml LPC (leupeptin/pepstatin/chymostatin) and 1 mM pefabloc). Cells were resuspended in lysis buffer and bead beat twice for 20 seconds. Extracts were incubated for 30 min with IgG-coupled Dynabeads (Invitrogen), which bind to Lid1-TAP. The immunoprecipitated complexes were washed three times with lysis buffer and then analyzed by immunoblotting with sheep anti-GFP antibody and sheep anti-Mad2 antibody.

Mad1-TAP - Mad2 interaction

Protein extracts were prepared from cycling cells expressing SZZ-tagged Mad1. Immunoprecipitations were carried out as described above with the following modifications. No phosphatase inhibitors were added to the lysis buffer. Immunoprecipitated complexes were analyzed by immunoblotting with a sheep anti-Mad2 antibody and Peroxidase-Anti Peroxidase (Sigma) antibody to detect Mad1-TAP.

Mph1-SZZ kinase purification

Cycling cells expressing Mph1-SZZ were pelleted, washed once with ice-cold water, frozen and ground in liquid nitrogen. For protein extraction the cell powder was resuspended in twice the cell powder weight 2 x Hyman lysis buffer (100 mM bis-Tris propane, 200 mM KCl, 10 mM EGTA, 10 mM EDTA, 20 % glycerol, protease inhibitors (1 mM pefablock, 10 µg/ml leupeptin/pepstatin/chymostatin), TritonX-100 (to 1 %) added and then sonicated for 30 sec. Lysed cells were centrifuged 10 min, 3000 g to remove cell debris. The remaining supernatant was then filtered through a 25 mm GD/X syringe filter (Whatman), pore size 2.6 mM and a 25 mm GD/X syringe filter (Whatman), pore size 1.6 mM. The clarified lysate was incubated with IgG-coupled dynabeads (Invitrogen) for 30 min. Beads were washed 3 times with 1 x Hyman buffer and then 3 times with 1 x Hyman buffer (+ 1 mM DTT, 0.1% Tween-20) in batch. The purified protein was cleaved off the beads using 100 units AcTEV protease (Invitrogen) overnight. The supernatant was then transferred to a new tube and the IgG-coupled Dynabeads washed once with 1 ml 1 x Hyman buffer (+ 1 mM DTT, 0.1 % Tween-20). The supernatant containing the cleaved protein was incubated with S-protein agarose beads (Novagen) for 3 hours. S-protein agarose beads were then washed five times with 1 x Hyman buffer.

***In vitro* kinase assay:**

Purified Mph1 kinase coupled to S-protein agarose beads was washed twice with 1 x kinase buffer (50 mM Hepes, pH 7.5, 10 mM $MgCl_2$, 0.5 mM DTT). 25 µl of kinase reaction buffer (12.5 µl 2x kinase buffer (100 mM Hepes, pH7.5, 20 mM $MgCl_2$, 1 mM DTT), 0.5 µl P^{32} gamma-ATP, 0.5 µl 1 mM ATP, made up with substrate in a final volume of 25 µl was then added to the beads. Reactions were typically carried out with 1.5 µg substrate/recombinant protein. The reaction was incubated at 30°C for 30 mins. Cold kinase assays were carried out with 100 mM ATP and further analysed by mass spectrometry or by immunoblotting with phosphoantibodies generated against phosphorylated residues S92 (Covance), S187, S189 (Eurogentec) of Mad2.

Mass-spectrometry

Protein digestion and Phosphopeptide enrichment

Proteins were electrophoresed into NovexNuPAGE 4-12% Bis-Tris gels (Invitrogen). Proteins were stained with a colloidal blue kit (Invitrogen). Proteins were excised and standard trypsin digestion procedure carried out as described previously [6]. The supernatant of samples for MS analysis without phosphopeptide enrichment were loaded onto StageTips [7]. Supernatants of samples for phosphopeptide enrichment were removed and peptides further extracted from the gel using 3% TFA/30% ACN solution followed by 100% ACN. Before enrichment approximately 3 mg TiO_2 beads were pre-incubated in 20 µl of 85 mg/ml lactic acid in 80% ACN/0.1%TFA. Pre-incubated bead mixture was added to the peptide mixture and incubated for 1 h at room temperature. After washing the beads once with 10% ACN/0.1% TFA, and twice with 80% ACN/0.1% TFA, peptides were eluted with 2% ammonium hydroxide in 40% ACN (pH 10.5). Eluate was concentrated to 100 µl and 100 µl of 2% TFA was added. All samples were loaded onto StageTips and stored at -20ºC for MS analysis.

Nano-LC-MS/MS and Data analysis

An LTQ-Orbitrap (Thermo Scientific), 1200 series nanoflow HPLC pump (Agilent Technologies) and HTC PAL auto sampler (CTC Analytics) were used for LC-MS/MS analysis. Mobile phases were (A) 5% acetonitrile, 0.5% acetic acid and (B) 99.5% acetonitrile, 0.5% acetic acid. A flow rate of 300 nl/min was used with a spray voltage of 1.8 kV. Analysis was done using either a two-step linear gradient of 0%-20% B in 35 min, 20%-80% B in 4 min and 80% B for 2 min or a two-step linear gradient of 0%-20% B in 75 min, 20%-80% B in 13 min and 80% for 10 min. A column needle was prepared by packing 3 µm Reprosil C18 materials (Dr. Maisch, Germany) into Pico Tip Emitter silica tips (8±1µm, New Objectives) under the pressure of nitrogen [8]. Each cycle consisted of one full MS scan acquired in the Orbitrap analyser, followed by MS/MS of the six most abundant peptides in the ion trap.

Database searching

DTAsupercharge (V1.18) was used to create peak lists from raw data. Peak lists were then used within Mascot daemon (V2.2.0) to search against the UniProt/SwissProt *Schizosaccharomyces pombe* database. Search parameters were set to: precursor mass tolerance of 10 ppm, fragment ion mass tolerance to 0.8 Da, enzyme as trypsin, allowing 3 missed cleavages. Carboamidomethylation of cysteine was set as a fixed modification, with oxidation of methionines, phosphorylation of serine, threonine, and tyrosine as variable modifications.

Supplemental References:

1. Gregan, J., Riedel, C.G., Pidoux, A.L., Katou, Y., Rumpf, C., Schleiffer, A., Kearsey, S.E., Shirahige, K., Allshire, R.C., and Nasmyth, K. (2007). The kinetochore proteins Pcs1 and Mde4 and heterochromatin are required to prevent merotelic orientation. Curr Biol *17*, 1190-1200.
2. West, R.R., Malmstrom, T., and McIntosh, J.R. (2002). Kinesins klp5(+) and klp6(+) are required for normal chromosome movement in mitosis. J Cell Sci *115*, 931-940.
3. Sironi, L., Mapelli, M., Knapp, S., De Antoni, A., Jeang, K.T., and Musacchio, A. (2002). Crystal structure of the tetrameric Mad1-Mad2 core complex: implications of a 'safety belt' binding mechanism for the spindle checkpoint. Embo J *21*, 2496-2506.
4. Keeney, J.B., and Boeke, J.D. (1994). Efficient targeted integration at leu1-32 and ura4-294 in Schizosaccharomyces pombe. Genetics *136*, 849-856.
5. Bernard, P., Hardwick, K., and Javerzat, J.P. (1998). Fission yeast bub1 is a mitotic centromere protein essential for the spindle checkpoint and the preservation of correct ploidy through mitosis. Journal of Cell Biology *143*, 1775-1787.
6. Maiolica, A., Cittaro, D., Borsotti, D., Sennels, L., Ciferri, C., Tarricone, C., Musacchio, A., and Rappsilber, J. (2007). Structural analysis of multiprotein complexes by cross-linking, mass spectrometry, and database searching. Mol Cell Proteomics *6*, 2200-2211.
7. Rappsilber, J., Ishihama, Y., and Mann, M. (2003). Stop and go extraction tips for MALDI, nanoelectrospray and LC/MS sample pretreatment in proteomics. Anal Chem *75*, 663-670.
8. Ishihama, Y., Rappsilber, J., Andersen, J.S., and Mann, M. (2002). Microcolumns with self-assembled particle frits for proteomics. J Chromatogr A *979*, 233-239.
